# Comparative proteomic analysis reveals that exogenous 6-benzyladenine (6-BA) improves the defense system activity of waterlogged summer maize

**DOI:** 10.1186/s12870-020-2261-5

**Published:** 2020-01-29

**Authors:** Juan Hu, Baizhao Ren, Shuting Dong, Peng Liu, Bin Zhao, Jiwang Zhang

**Affiliations:** State Key Laboratory of Crop Biology and College of Agronomy, Shandong Agricultural University, Taian, Shandong 271018 People’s Republic of China

**Keywords:** Maize (*Zea mays* L.), Leaf, Proteomic, Tandem mass tags, Waterlogging; 6-benzyladenine

## Abstract

**Background:**

Exogenous 6-benzyladenine (6-BA) could improve leaf defense system activity. In order to better understand the regulation mechanism of exogenous 6-benzyladenine (6-BA) on waterlogged summer maize, three treatments including control (CK), waterlogging at the third leaf stage for 6 days (V3–6), and application of 100 mg dm^− 3^ 6-BA after waterlogging for 6 days (V3–6-B), were employed using summer maize hybrid DengHai 605 (DH605) as the experimental material. We used a labeling liquid chromatography-based quantitative proteomics approach with tandem mass tags to determine the changes in leaf protein abundance level at the tasseling stage.

**Results:**

Waterlogging significantly hindered plant growth and decreased the activities of SOD, POD and CAT. In addition, the activity of LOX was significantly increased after waterlogging. As a result, the content of MDA and H_2_O_2_ was significantly increased which incurred serious damages on cell membrane and cellular metabolism of summer maize. And, the leaf emergence rate, plant height and grain yield were significantly decreased by waterlogging. However, application of 6-BA effectively mitigated these adverse effects induced by waterlogging. Compared with V3–6, SOD, POD and CAT activity of V3–6-B were increased by 6.9, 12.4, and 18.5%, LOX were decreased by 13.6%. As a consequence, the contents of MDA and H_2_O_2_ in V3–6-B were decreased by 22.1 and 17.2%, respectively, compared to that of V3–6. In addition, the leaf emergence rate, plant height and grain yield were significantly increased by application of 6-BA. Based on proteomics profiling, the proteins involved in protein metabolism, ROS scavenging and fatty acid metabolism were significantly regulated by 6-BA, which suggested that application of 6-BA exaggerated the defensive response of summer maize at proteomic level.

**Conclusions:**

These results demonstrated that 6-BA had contrastive effects on waterlogged summer maize. By regulating key proteins related to ROS scavenging and fatty acid metabolism, 6-BA effectively increased the defense system activity of waterlogged summer maize, then balanced the protein metabolism and improved the plant physiological traits and grain yield.

## Background

Global warming has been unequivocally confirmed with many unprecedented observed changes such as increasing concentrations of greenhouse gases, warming of atmosphere, and extreme rainfall events over decades to millennia [1]. In China, the average surface temperature has increased by 1.1 °C, and the increase rate of temperature has reached 0.22 °C/10a over the last five decades [2]. The spatial and temporal distribution of rainfall has tended toward extremes and adverse climatic events, including waterlogging, drought, heat injury, low temperature, and freezing damage, are expected [3]. Such extreme events have done untold damages to environment, agricultural production, and long-term prospects of economy [4–7]. The frequency and intensity of extreme rainstorm events has increased in most parts of China since the 1980s, and the number of rainstorm days in the south of the Yangtze River and western, northern, and central parts of Henan Province has increased significantly since the 1990s [8]. Moreover, rainstorms and extreme precipitation events have tended to increase in south of 34°N [9]. Rainstorms are the most typical cause of waterlogging, which cause serious grain yield losses due to its paroxysmal and unpredictable nature [10]. Thus, the maize production system in Huang-Huai-Hai Plain of China faces great challenges and risks against a backdrop of changing climate and increasing numbers of disastrous events. During the whole life cycle of maize production in Huang-Huai-Hai Plain, the frequency of waterlogging is as high as 30%, especially during seedling to jointing and jointing to tasseling stages, which damages plant growth and increases grain yield loss significantly [9].

Maize is generally susceptible to waterlogging which may occur at different growth stages. Previous studies showed that waterlogging at different growth stages incurred diverse effects on the growth and yield of summer maize [11, 12]. To address waterlogging stress, plants would initiate a series of stress defensing processes going along with a wide-ranging changes of cell activities in plants. Plant defense system plays a key role in protecting plant from damaging under stresses [13]. As the antioxidant defense system was damaged under abiotic stresses, a significant increase in the accumulation of ROS in maize leaves was triggered which resulted in lipid peroxidation and membrane permeability [14]. At first, under adverse conditions (for examples, drought, salinity, cold, shade and so on), reactive oxygen species (ROS) are being generated as a secondary messenger to initiate subsequent defense reaction in plants which plays important roles in plant defense response to abiotic stress [15]. However, with the prolongation of stress duration, the ROS scavenging system was upset which disturbed the balance between production and quenching of ROS resulting in oxidative damages [16–20]. Antioxidant enzymes could effectively reduce the ROS damage on plants which activity may be directly related with plant tolerance to abiotic stresses [15].

6-Benzyladenine (6-BA) is a synthetic cytokinin (CTK)-like plant growth regulator that can significantly increase CTK levels in plants, of which levels are dramatically diminished under stress. CTKs are important growth-promoting compounds involved in inhibiting and scavenging active oxygen radicals, delaying leaf senescence. It has been reported that CTK improved the growth of plants exposed to stress by increasing superoxide dismutase activity and mitigating lipid peroxidation to maintain the balance between the production and scavenging of active oxygen radicals [21–23]. Applying 6-BA was conducive to minimize adverse effects of environmental stress such as drought, salt, low-temperature, and waterlogging stress [23–25]. In our previous studies, application of 6-BA effectively improved summer maize growth and increased the grain yield of summer maize under waterlogging stresses [26]. However, many studies have assessed the effects of 6-BA on plant growth, while only a few studies have investigated the regulation of molecular mechanism of exogenous 6-BA on the growth and development of waterlogged summer maize.

The proteome is the sum of all proteins expressed via gene transcription. Proteomics is a technique for studying the proteome to better represent life true characteristics at a deep level. It is significant to explore and discover the laws regulating biological activities, important physiological and pathological phenomena [27]. Proteomic analyses provide new insights to explore the potential of enhancing stress tolerance [28–30]. The changes of physiological metabolic processes at different plant stages can be studied by proteome analysis, because high-throughput proteomics studies cannot only reveal the mechanism of stress-related metabolic responses, but also reflect the specificity of different stress factors [30, 31]. Therefore, proteomics has attracted much academic attention. In particular, the different expression patterns of plant proteome provide insights into the regulation mechanisms and protective strategies of plants under different environmental stresses [32].

So far, quite a few studies have investigated the mechanism of waterlogging on summer maize, however, investigating the defense response mechanism of 6-BA on waterlogged summer maize at a level of proteins is still wanted. To mitigate this knowledge gap, a comparative proteomics study was applied to investigate the molecular mechanisms of 6-BA on waterlogged summer maize. Proteins related to plant defense system activity whose abundance was regulated by 6-BA were identified. Combining with the physiological traits, this study contributed to a better understanding of how exogenous 6-benzyladenine (6-BA) regulated the defense system activity in waterlogged summer maize.

## Results

### Plant growth and grain yield of summer maize

**Fig. 1:**
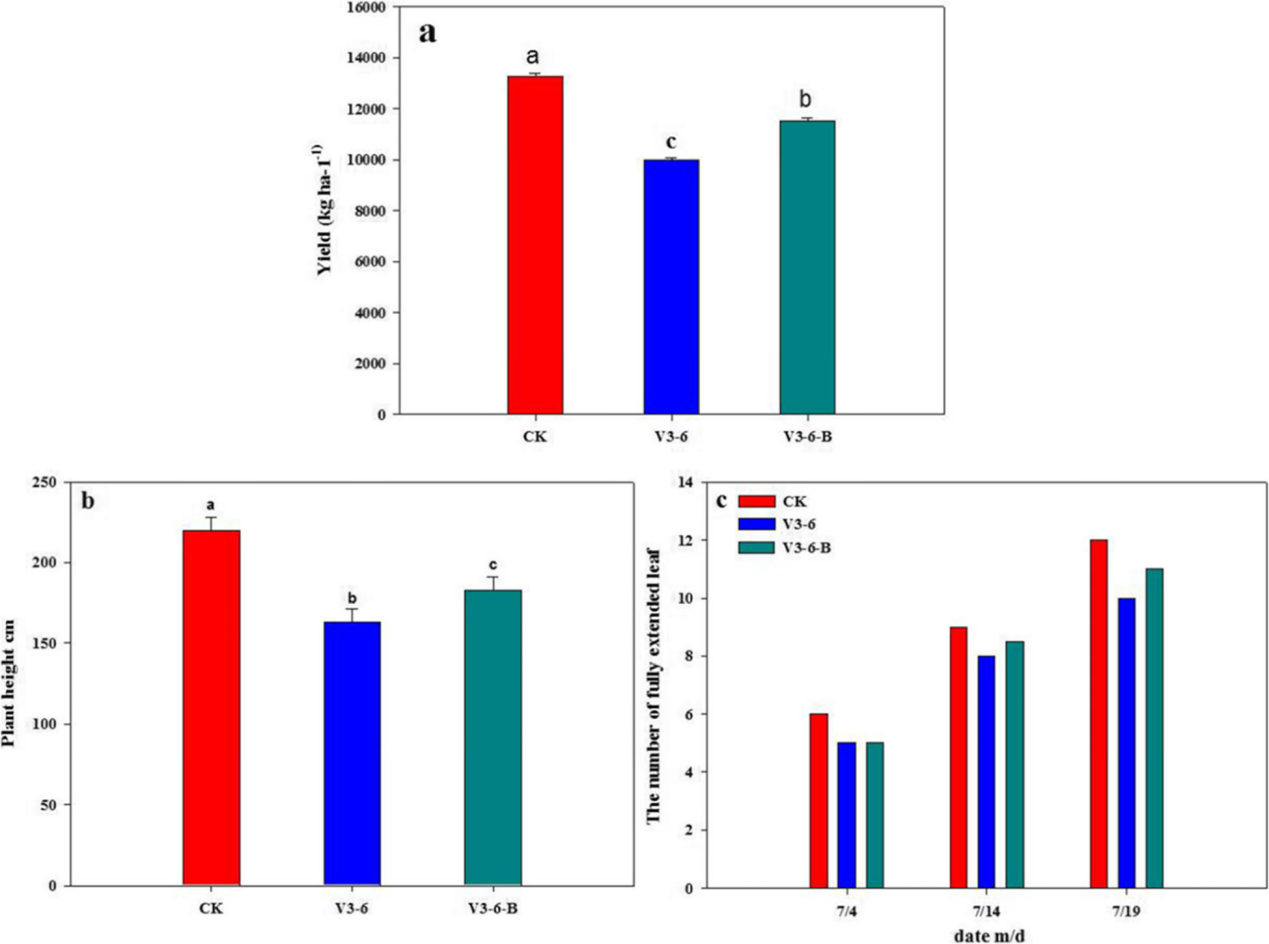
The plant growth and grain yield of summer maize, **a** The grain yield (kg ha^-1^) of summer maize; **b** The plant height (cm) of sumer maize; **c** The number of fully extended leaf. Data are means of three replicate experiments. Symbols followed by different letters denote statistical difference at *P* < 0.05 using ANOVA followed by LSD test

The field experiment results showed that waterlogging significantly impeded the plant growth by decreasing leaf emergence rate and plant height. At VT, the plant height of waterlogging treatment (V3–6) was decreased by 25.9%, compared to that of CK. However, spraying 6-BA was conducive to mitigate the decrease of plant height of waterlogged maize. The plant height of V3–6-B was increased by 12.3%, compared to that of V3–6. The leaf emergence rate was delayed by waterlogging, the V6, V9, V12 growth and development stages were delayed by 1, 2, 2 days in V3–6, respectively, compared with CK. In V3–6-B, the V6, V9, V12 growth and development stages were all delayed by 1 days, compared with that of CK (Fig. 1).

### ROS scavenging system

**Fig. 2:**
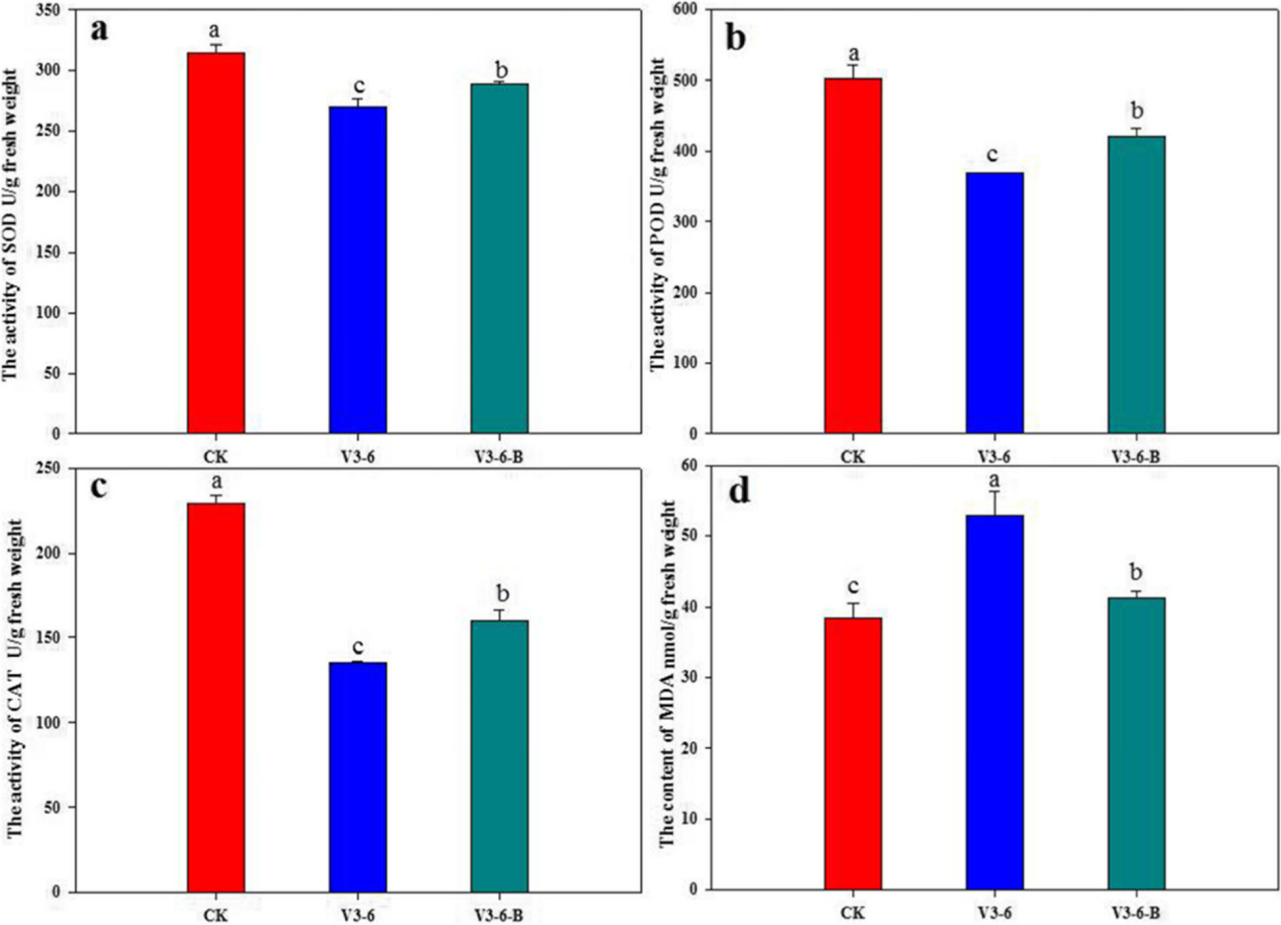
The enzymes (SOD, POD, CAT) activity and MDA content in different treatments, **a** The activity of SOD (U/g fresh weight) in summer maize; **b** The activity of POD (U/g fresh weight) in summer maize; **c** The activity of CAT (U/g fresh weight) in summer maize; **d** The content of MDA (nmol/g fresh weight)  in summer maize. Data are means of three replicate experiments. Symbols followed by different letters denote statistical difference at *P* < 0.05 using ANOVA followed by LSD test

The activities of SOD, POD, and CAT were significantly decreased after waterlogging. The reduction in the activities of SOD, POD and CAT were around 14.0, 16.3 and 41.0%, respectively, compared to those of CK. The MDA content was increased by 38% in V3–6, compared to that of CK. However, application of 6-BA alleviated waterlogging stress on the activities of antioxidative enzymes. Applying 6-BA resulted in significant and substantial increases in SOD (around 6.9%), POD (around 12.4%), and CAT (around 18.5%), compared to those of the waterlogging treatment. And also, application of 6-BA alleviated waterlogging stress on MDA content, with 22.1% decrease of MDA content in V3–6-B, compared to that of V3–6 (Fig. 2).

### LOX activity and H2O2 content

**Fig. 3:**
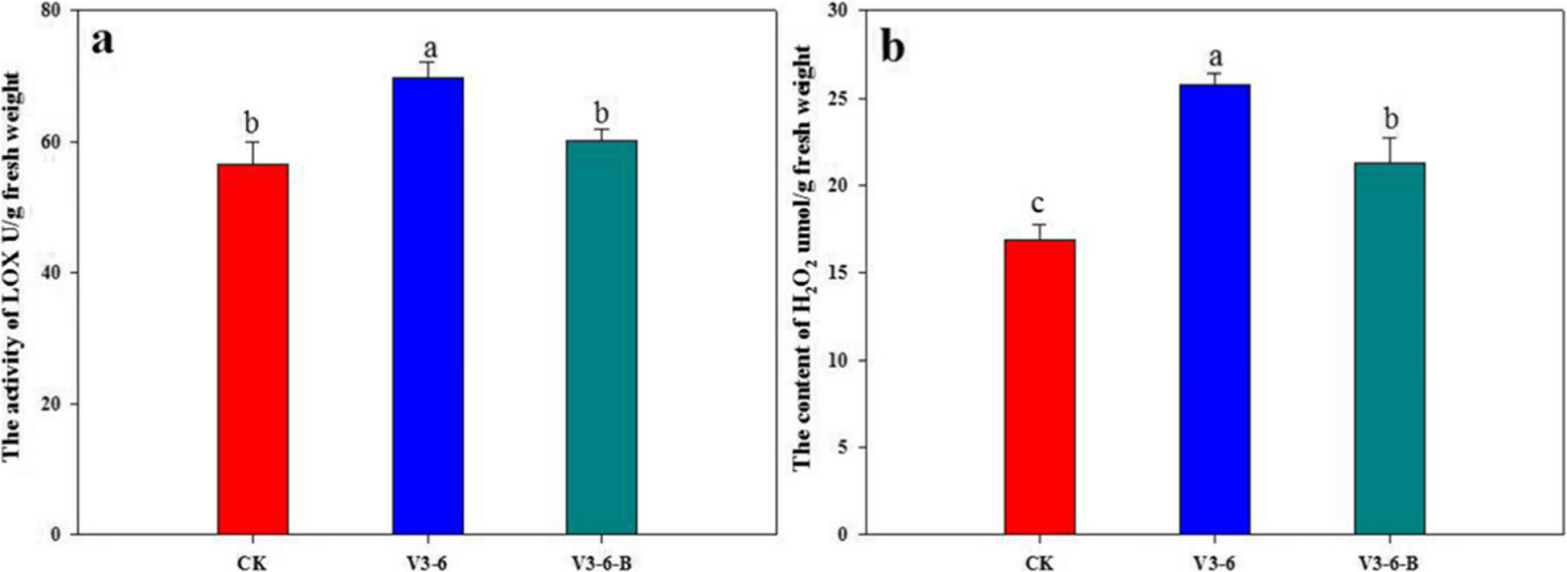
The LOX activity and H_2_O_2_ content in different treatments, **a** The activity of LOX (U/g fresh weight); **b** The content of H_2_O_2_ (umol/g fresh weight). Data are means of three replicate experiments. Symbols followed by different letters denote statistical difference at *P* < 0.05 using ANOVA followed by LSD test

In this study, the LOX activity was increased by 23.4% after waterlogging, however, this increase was effectively inhibited by application of 6-BA. In addition, the H_2_O_2_ content in V3–6 treatment was about 52.3% higher than in CK, while that of V3–6-B was about 17.2% lower than that of V3–6 (Fig. 3).

### Identification of differentially accumulated proteins

The result of quality detection by mass spectrometry showed that the peptide mass error central distributes in below 10 ppm scopes and most peptide distributed in 8–20 amino acid residues (Additional file 1: Figure S1). In addition, the results of Pearson’s correlation of quantitation among treatments also indicated that our samples reached the requirements for further study (Additional file 1: Figure S2). In total, 5322 leaf proteins were detected, and 4437 were quantified, using the criteria that proteins with a fold-change ≥1.5 were considered as differentially abundant proteins. One hundred fifty two and 28 leaf proteins were identified as significantly up-regulated and down-regulated proteins under waterlogging stress (V3–6), 48 and 67 proteins were identified as significantly up-regulated and down-regulated proteins in V3–6-B treatment, respectively, and 222 proteins were differentially abundant between V3–6 and V3–6-B (Additional file 1: Figure S3). Among the differentially accumulated proteins, about 4% were involved in antioxidant activity, 10% were involved in responding to stimulus, and 30% were related to metabolic process, these proteins were mostly related with plant defense system (Additional files 2 and 3).

### Bioinformatic analysis of differentially abundant proteins

**Fig. 4:**
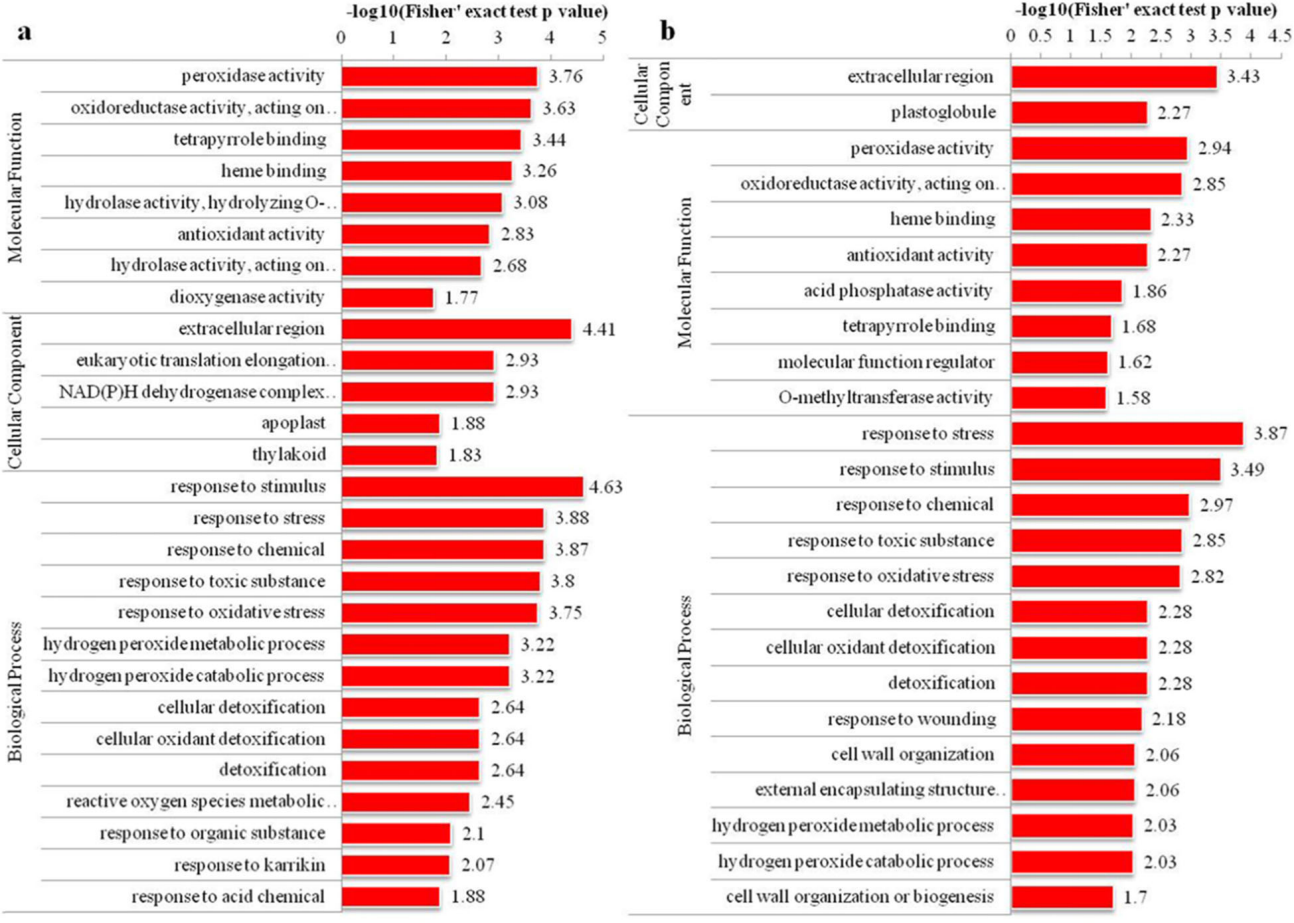
**a** GO enrichment analysis of differentially abundant proteins between V3–6 and CK, **b** GO enrichment analysis of differentially abundant proteins between V3–6-B and CK

GO annotation was performed to identify the significantly enriched GO functional groups of differentially abundant proteins. Comparing with CK, The up-accumulated proteins of V3–6 and V3–6-B treatments were strongly enriched in defense-related proteins including “plant response to stress”, “response to toxic substance”, “response to oxidative stress”, “response to stimulus”, “hydrogen peroxide metabolic process” and mainly had functions in peroxidase activity, tetrapyrrole binding and oxidoreductase activity. These proteins played key roles in protecting plant from damages (Fig. 4).

### Differentially abundant proteins involved in ROS scavenging system

Proteomic data showed that proteins involved in ROS scavenging system such as peroxidase (A0A1D6E530, B4FBH0, B4FKV6, B4FVT1, C0HHA6) were significantly up-accumulated in V3–6 treatment compared with CK. While, L-ascorbate peroxidase (A0A1D6QMU5), Glutathione peroxidase (C0P3R8) and APx3-Peroxisomal Ascorbate Peroxidase (B6TM55) were down-accumulated in V3–6 treatment compared with CK. However, the peroxidases mentioned above were down-accumulated in V3–6-B treatment compared with V3–6. And L-ascorbate peroxidase (A0A1D6QMU5), Glutathione peroxidase (C0P3R8) and APx3-Peroxisomal Ascorbate Peroxidase (B6TM55) were up but not significantly accumulated in V3–6-B treatment compared with V3–6 (Table 1).

**Table 1:** Differentially abundant proteins involved in ROS scavenging system

Protein accession	Protein description	Score	Coverage [%]	Peptides	PSMs	Unique peptides	V3–6/CK Ratio	V3–6/CK *P* value	V3–6-B/CK Ratio	V3–6-B/CK *P* value	V3–6-B/V3–6 Ratio	V3–6-B/V3–6 *P* value
A0A1D6E530	Peroxidase OS = *Zea mays* GN = ZEAMMB73_Zm00001d002899	160	47.2	9	15	9	1.57	0.0006	1.49	0.000737	0.95	0.115
A0A1D6HAE3	L-ascorbate peroxidase S chloroplastic/mitochondrial OS = Zea mays GN = ZEAMMB73_Zm00001d016802	288	37.2	16	40	13	1.15	0.00516	0.74	0.00000033	0.64	0.00000127
A0A1D6I3Z9	Putative cinnamyl alcohol dehydrogenase 6 OS = Zea mays GN = ZEAMMB73_Zm00001d020402	101	48.4	5	19	5	1.20	0.00048	1.23	0.000218	1.02	0.258
A0A1D6N0K3	Peroxidase OS = Zea mays GN = ZEAMMB73_Zm00001d042022	79	25.2	8	15	8	1.28	0.0003	0.83	0.000856	0.65	0.0000788
A0A1D6QMU5	L-ascorbate peroxidase 3 peroxisomal OS = Zea mays GN = ZEAMMB73_Zm00001d053223	123	38.3	13	24	11	0.69	0.00014	0.78	0.000622	1.13	0.000801
A5H8G4	Peroxidase 1 OS = Zea mays GN=PER1	131	34.6	10	23	8	1.22	0.00024	1.10	0.0147	0.90	0.00762
B4F7T9	Peroxidase OS = Zea mays GN = ZEAMMB73_Zm00001d023899	75	25.7	7	12	6	1.31	0.00068	1.15	0.024	0.88	0.0117
B4FBH0	Peroxidase OS = Zea mays GN = ZEAMMB73_Zm00001d024734	89	26.3	7	9	7	2.76	2.3E-07	1.89	9.21E-07	0.69	0.0000167
B4FKV6	Peroxidase OS = Zea mays GN = ZEAMMB73_Zm00001d014341	27	9.9	4	5	4	1.65	0.00014	0.86	0.0186	0.52	0.0000046
B4FVT1	Peroxidase OS = Zea mays GN = ZEAMMB73_Zm00001d037550	29	10.4	4	7	3	1.75	0.0012	1.01	0.547	0.58	0.0000226
B6TM55	APx1-Cytosolic Ascorbate Peroxidase OS = Zea mays GN = ZEAMMB73_Zm00001d047757	152	61.6	13	40	3	0.84	0.00514	0.90	0.0461	1.07	0.024
C0HHA6	Peroxidase OS = Zea mays GN = ZEAMMB73_Zm00001d008173	8	2.6	1	1	1	2.50	8.5E-05	1.72	0.00208	0.69	0.00354
C0P3R8	Glutathione peroxidase OS = Zea mays GN = ZEAMMB73_Zm00001d037079	65	35.2	7	19	7	0.78	2.4E-05	0.85	0.000496	1.09	0.0039
C4J6E4	Peroxidase OS = Zea mays GN = ZEAMMB73_Zm00001d022456	39	34.6	4	7	4	0.94	0.264	0.67	0.00502	0.71	0.0136
K7VCN5	Peroxidase OS = Zea mays GN = ZEAMMB73_Zm00001d009373	146	34	7	7	7	1.44	0.00074	1.28	0.00106	0.89	0.0583

### Differentially abundant proteins involved in fatty acid metabolism

**Fig. 5:**
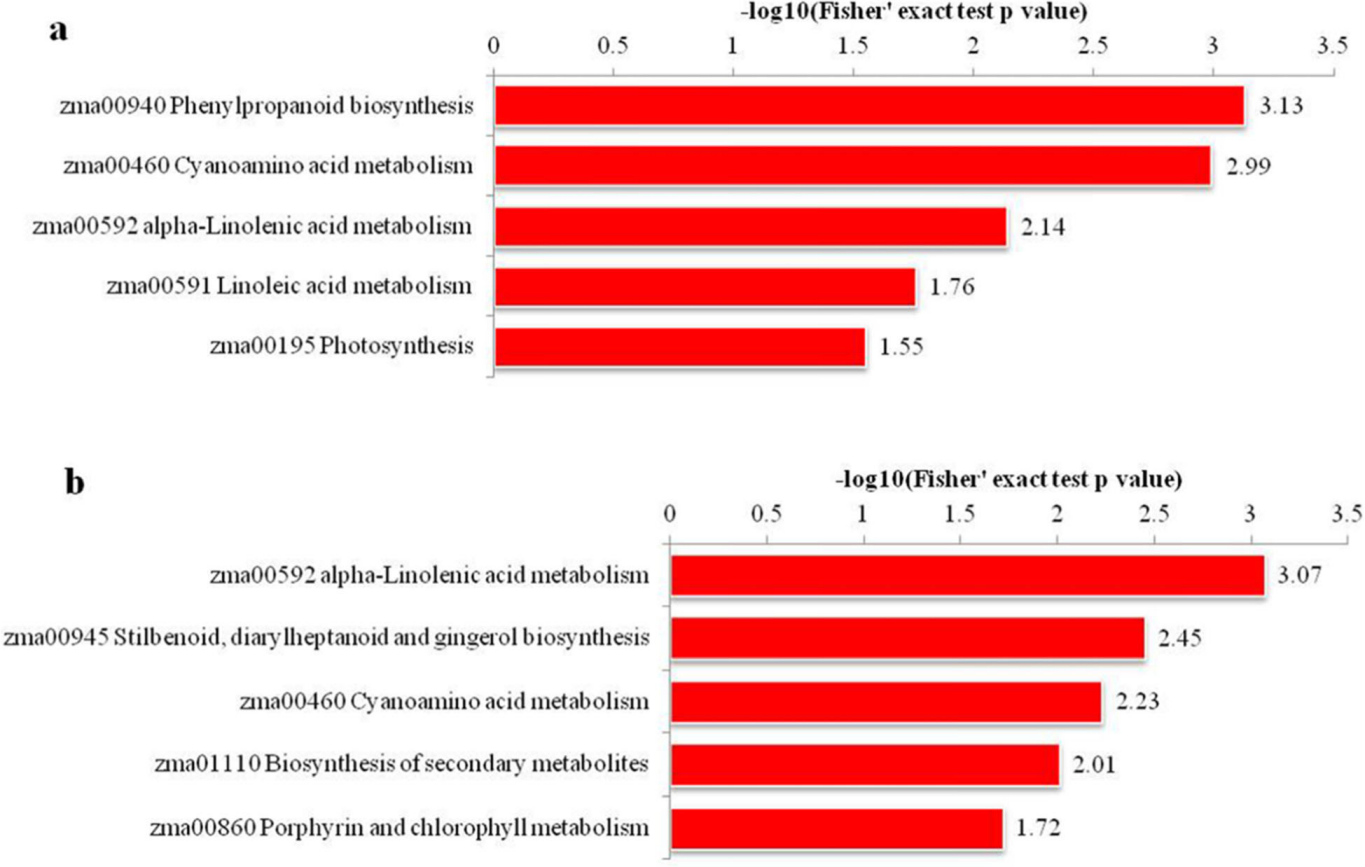
**a** KEGG pathway cluster analysis of differentially abundant proteins between V3–6 and CK, **b** KEGG pathway cluster analysis of differentially abundant proteins between V3–6-B and CK

Proteins that up-accumulated in V3–6-B and V3–6 compared with CK were strongly enriched in Linoleic acid metabolism and alpha- Linolenic acid metabolism, including Alpha-dioxygenase 1(H9BG22 and A0A1D6P493), Lipoxygenase (Q9LKL4). In addition, proteins involved in fatty acid metabolism such as Acyl carrier protein (B6UHG1, B6SJF5, B4FDG2), Diphosphonucleotide phosphatase1 (C0PES7) and Triglyceride lipases (A0A1D6JA03) were up-accumulated in V3–6, but down-accumulated in V3–6-B. However, Fatty acid desaturase8 were down-regulated in V3–6 and V3–6-B (Table 2; Fig. 5).

**Table 2:** Differentially abundant proteins involved in fatty acid metabolism

Protein accession	Protein description	Score	Coverage [%]	Peptides	PSMs	Unique peptides	V3–6/CK Ratio	V3–6/CK *P* value	V3–6-B/CK Ratio	V3–6-B/CK *P* value	V3–6-B/V3–6 Ratio	V3–6-B/V3–6 *P* value
A0A1D6G949	Protein FATTY ACID EXPORT 3 chloroplastic OS = Zea mays GN = ZEAMMB73_Zm00001d012504	69	14.6	4	5	1	1.22	0.041	0.93	0.621	0.77	0.192
A0A1D6J5I4	Fatty acid biosynthesis1 OS = Zea mays GN = ZEAMMB73_Zm00001d025201	41	39.7	7	10	4	1.47	0.00066	1.34	0.00158	0.92	0.00964
A0A1D6JA03	Triglyceride lipases OS = Zea mays GN = ZEAMMB73_Zm00001d025827	20	2.5	1	1	1	1.90	0.0115	0.72	0.139	0.38	0.000195
A0A1D6JQF2	Lipoxygenase OS = Zea mays GN = ZEAMMB73_Zm00001d027893	8	2.5	1	1	1	1.62	0.00736	0.90	0.314	0.56	0.00432
A0A1D6JZ55	Fatty acid desaturase8 OS = Zea mays GN = ZEAMMB73_Zm00001d028742	14	3.8	2	2	2	0.80	0.00052	0.84	0.0041	1.05	0.245
A0A1D6P493	Alpha-dioxygenase 1 OS = Zea mays GN = ZEAMMB73_Zm00001d046636	12	5.2	1	1	1	1.60	0.00476	1.87	0.001	1.17	0.111
B4FDG2	Acyl carrier protein OS = Zea mays GN = ZEAMMB73_Zm00001d041701	14	17.1	2	6	2	1.94	0.00016	0.93	0.343	0.48	0.000521
B4FVE3	Farnesyl diphosphate synthase3 OS = Zea mays GN = ZEAMMB73_Zm00001d043727	20	8.2	3	5	3	2.06	2.2E-05	0.50	0.000117	0.24	0.00000415
B6SJF5	Acyl carrier protein OS = Zea mays GN = ZEAMMB73_Zm00001d032019	7	8.4	1	1	1	1.96	2.4E-06	0.72	0.000344	0.37	0.0000025
B6UHG1	Acyl carrier protein OS = Zea mays GN = ZEAMMB73_Zm00001d019186	7	6.8	1	3	1	1.37	0.00112	0.86	0.0269	0.63	0.000222
C0PES7	Diphosphonucleotide phosphatase1 OS = Zea mays GN = ZEAMMB73_Zm00001d023698	186	30.7	10	21	10	1.34	4.5E-06	0.87	0.00128	0.65	0.00000418
H9BG22	Alpha-dioxygenase 1 OS = Zea mays GN = ZEAMMB73_Zm00001d035854	96	21	11	16	11	1.56	5.9E-05	1.65	0.000024	1.06	0.0474
Q9LKL4	Lipoxygenase OS = Zea mays GN = LOX	188	26.5	20	25	20	1.93	0.00822	1.65	0.0141	0.85	0.000176

### Differentially abundant proteins involved in proteins metabolism

**Fig. 6:**
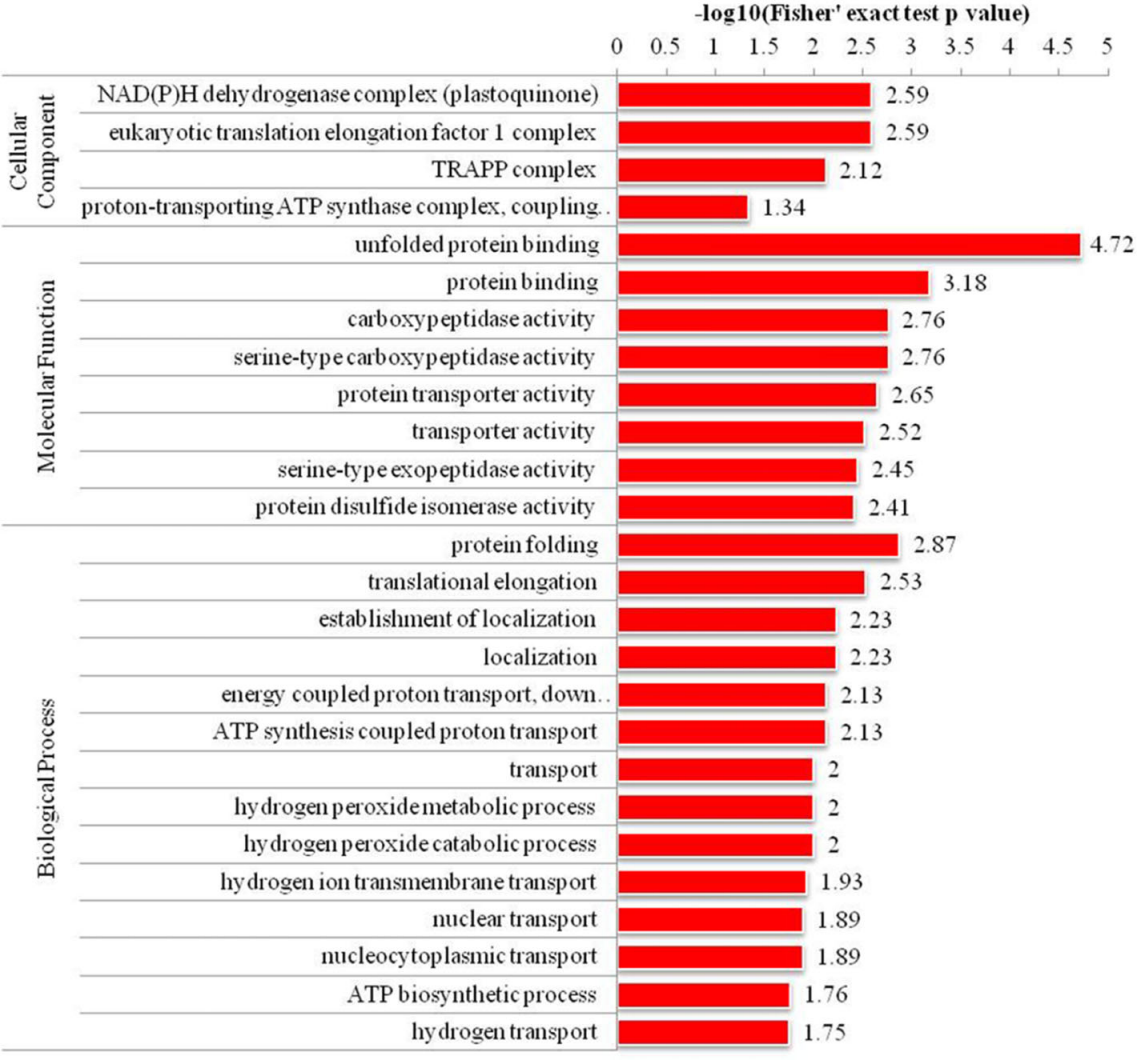
GO enrichment analysis of differentially abundant proteins between V3–6-B and V3–6

Compared with V3–6, the down-accumulated proteins of V3–6-B were enriched into 26 functional groups, of which molecular functions and biological processes accounted for 8 and 14 GO terms, respectively. Interestingly, the most up-accumulated proteins were involved in proteins metabolism including “unfolded protein binding”, “protein binding”, “protein transporter activity”, “protein disulfide isomerase activity”, and “protein folding” (Table 3; Fig. 6).

**Table 3:** Differentially abundant proteins involved in proteins metabolism

Protein accession	Protein description	Score	Coverage [%]	Peptides	PSMs	Unique peptides	V3–6/CK Ratio	V3–6/CK *P* value	V3–6-B/CK Ratio	V3–6-B/CK *P* value	V3–6-B/V3–6 Ratio	V3–6-B/V3–6 *P* value
A0A1D6E942	30S ribosomal protein 3 chloroplastic OS = Zea mays GN = ZEAMMB73_Zm00001d003411	19	16	3	6	3	1.51	5.8E-05	0.86	0.0026	0.57	0.00000432
A0A1D6ES19	Heat shock protein 90–2 OS = Zea mays GN = ZEAMMB73_Zm00001d006008	16	36.4	21	27	2	1.63	0.00058	0.98	0.765	0.60	0.000603
A0A1D6GG02	DnaJ protein OS = Zea mays GN = ZEAMMB73_Zm00001d013111	12	30.7	12	17	2	1.13	0.0477	0.71	0.00432	0.63	0.00202
A0A1D6GK64	Heat shock 70 kDa protein 6 chloroplastic OS = Zea mays GN = ZEAMMB73_Zm00001d013507	44	51.2	36	125	2	1.17	0.0416	0.72	0.0186	0.62	0.00404
A0A1D6GTD7	26S proteasome non-ATPase regulatory subunit 13 homolog A OS = Zea mays GN = ZEAMMB73_Zm00001d014436	49	20.9	6	6	3	1.51	0.0122	1.02	0.895	0.67	0.0311
A0A1D6GWT5	Calcium-binding EF hand family protein OS = Zea mays GN = ZEAMMB73_Zm00001d014862	16	1.7	2	2	2	1.31	0.0191	0.85	0.0303	0.65	0.00178
A0A1D6GZM9	RING/FYVE/PHD zinc finger superfamily protein OS = Zea mays GN = ZEAMMB73_Zm00001d015152	6	10.8	1	1	1	2.35	0.00596	0.63	0.00938	0.27	0.00328
A0A1D6GZY9	Cleavage and polyadenylation specificity factor (CPSF) A subunit protein OS = Zea mays GN = ZEAMMB73_Zm00001d015208	65	5.6	7	9	7	1.43	0.00056	0.82	0.0192	0.57	0.00026
A0A1D6H4K9	Nucleic acid-binding OB-fold-like protein OS = Zea mays GN = ZEAMMB73_Zm00001d015917	16	16.2	2	2	2	1.57	1.8E-05	1.10	0.0534	0.71	0.000604
A0A1D6H5B6	50S ribosomal protein L21 chloroplastic OS = Zea mays GN = ZEAMMB73_Zm00001d016072	49	34	4	7	4	1.60	0.00016	0.82	0.0385	0.51	0.000382
A0A1D6HCE3	26S proteasome non-ATPase regulatory subunit 6-like protein OS = Zea mays GN = ZEAMMB73_Zm00001d017117	36	11.4	4	7	2	1.80	1.8E-06	0.93	0.182	0.52	0.000125
A0A1D6HG23	Uncharacterized protein OS = Zea mays GN = ZEAMMB73_Zm00001d017621	61	25.5	4	10	4	1.24	0.00136	0.73	0.00000299	0.59	0.0000366
A0A1D6HT76	Protein containing PDZ domain a K-box domain and a TPR region OS = Zea mays GN = ZEAMMB73_Zm00001d018901	159	37.6	9	21	9	1.62	1.1E-06	0.65	0.000341	0.40	0.00164
A0A1D6II13	Calcium-dependent lipid-binding (CaLB domain) family protein OS = Zea mays GN = ZEAMMB73_Zm00001d021952	27	6.5	2	2	1	1.22	0.0366	0.80	0.0167	0.66	0.00254
A0A1D6JX93	Peroxisomal nicotinamide adenine dinucleotide carrier OS = Zea mays GN = ZEAMMB73_Zm00001d028542	32	14.6	4	5	4	1.59	0.00092	1.00	0.976	0.63	0.000185
A0A1D6KA58	26S proteasome non-ATPase regulatory subunit 13 homolog A OS = Zea mays GN = ZEAMMB73_Zm00001d030126	24	15.6	5	5	2	1.52	7.5E-05	1.23	0.00482	0.81	0.00676
A0A1D6KE00	Thaumatin-like protein OS = Zea mays GN = ZEAMMB73_Zm00001d030694	204	21.5	18	25	9	1.29	0.00458	0.80	0.157	0.62	0.0219
A0A1D6KJP4	Peptidylprolyl isomerase OS = Zea mays GN = ZEAMMB73_Zm00001d031569	124	27.6	13	16	13	1.48	9.2E-07	0.82	0.00128	0.55	0.0000189
A0A1D6KSJ1	Putative prefoldin subunit 5 OS = Zea mays GN = ZEAMMB73_Zm00001d032649	10	13.8	1	1	1	1.65	0.00494	1.06	0.598	0.64	0.00206
A0A1D6L6U7	NmrA-like negative transcriptional regulator family protein OS = Zea mays GN = ZEAMMB73_Zm00001d034357	23	9.2	2	2	2	0.48	2.5E-08	0.53	0.0111	1.12	0.248
A0A1D6LBS9	Protein prenylyltransferase superfamily protein OS = Zea mays GN = ZEAMMB73_Zm00001d034833	17	11.2	2	3	2	1.45	0.00226	0.68	0.0489	0.47	0.00486
A0A1D6LIX0	Protein kinase superfamily protein with octicosapeptide/Phox/Bem1p domain OS = Zea mays GN = ZEAMMB73_Zm00001d035817	59	8.1	8	9	6	1.33	0.00606	0.82	0.0307	0.62	0.0012
A0A1D6M323	Ribosomal protein OS = Zea mays GN = ZEAMMB73_Zm00001d038084	236	42.3	15	35	14	2.01	1.7E-07	0.69	0.0059	0.34	0.00000192
A0A1D6MBB0	Nucleosome assembly protein 1 OS = Zea mays GN = ZEAMMB73_Zm00001d038851	25	7.5	2	3	1	1.89	2.9E-07	0.90	0.205	0.48	0.00578
A0A1D6MDC0	Protein SUPPRESSOR OF PHYA-105 1 OS = Zea mays GN = ZEAMMB73_Zm00001d039072	-2	0.7	1	1	1	1.40	0.224	0.82	0.553	0.59	0.0255
A0A1D6MUF1	Heat shock 70 kDa protein 6 chloroplastic OS = Zea mays GN = ZEAMMB73_Zm00001d041119	323	55	35	98	7	1.08	0.0124	0.69	0.0000396	0.64	0.0000363
A0A1D6MWV7	Importin subunit beta-1 OS = Zea mays GN = ZEAMMB73_Zm00001d041556	23	15.8	10	14	2	1.45	0.0107	0.78	0.0136	0.54	0.00206
A0A1D6NQK6	SMAD/FHA domain-containing protein OS = Zea mays GN = ZEAMMB73_Zm00001d044666	80	17.9	3	4	3	1.33	0.00962	0.73	0.00666	0.55	0.000518
A0A1D6PIZ9	Tubulin-tyrosine ligase OS = Zea mays GN = ZEAMMB73_Zm00001d048282	30	5.1	4	5	4	1.02	0.853	0.68	0.0255	0.66	0.0168
A0A1D6QK75	Heat shock protein 90–5 chloroplastic OS = Zea mays GN = ZEAMMB73_Zm00001d052809	34	11.9	8	10	3	1.40	0.0166	0.83	0.13	0.59	0.00038
B4F8Q2	Uncharacterized protein OS = Zea mays	42	40.6	25	55	2	1.28	0.00678	0.62	0.00126	0.48	0.00038
B4FCK9	60S ribosomal protein L22–2 OS = Zea mays GN = ZEAMMB73_Zm00001d022463	57	46.9	5	8	4	1.62	0.00148	0.89	0.196	0.55	0.000221
B4FI21	ARM repeat superfamily protein OS = Zea mays GN = ZEAMMB73_Zm00001d053003	161	36.5	13	17	3	1.10	0.1	0.64	0.000498	0.58	0.000156
B4FKB3	50S ribosomal protein L31 OS = Zea mays GN = ZEAMMB73_Zm00001d044130	10	8.5	1	5	1	1.43	0.00054	0.78	0.00418	0.55	0.0000366
B4FNC9	Small ubiquitin-related modifier OS = Zea mays GN=SUMO1a	77	48.5	6	9	6	1.30	0.00172	0.74	0.00122	0.57	0.000018
B4FNT1	Elongation factor 1-beta OS = Zea mays GN = ZEAMMB73_Zm00001d022134	48	22.3	4	7	4	1.65	0.00034	0.96	0.378	0.58	0.000083
B4FT63	Genomes uncoupled4-like protein OS = Zea mays GN = ZEAMMB73_Zm00001d023427	13	7.4	2	2	2	1.52	4E-06	0.69	0.0212	0.45	0.000141
B4FT80	Nucleic acid-binding OB-fold-like protein OS = Zea mays GN = ZEAMMB73_Zm00001d045607	8	8.4	1	1	1	1.60	0.00168	0.79	0.083	0.49	0.0028
B4FUA8	Calreticulin-3 OS = Zea mays GN = ZEAMMB73_Zm00001d012170	85	30.6	11	15	10	1.21	0.00336	0.68	0.0000216	0.56	0.0000245
B4FUZ5	30S ribosomal protein S1 OS = Zea mays GN = ZEAMMB73_Zm00001d047581	72	26.4	8	13	8	1.57	1.7E-05	1.00	0.919	0.64	0.000465
B4FVI4	60S acidic ribosomal protein P2–5 OS = Zea mays GN = ZEAMMB73_Zm00001d026578	8	9	1	1	1	2.14	0.00022	1.38	0.000382	0.65	0.00128
B6SLK4	Elongation factor 1-beta OS = Zea mays GN = ZEAMMB73_Zm00001d022513	100	38.8	8	18	8	2.01	1.9E-07	0.65	0.000197	0.32	0.00000202
B6T9G1	26S proteasome non-ATPase regulatory subunit 6 OS = Zea mays GN = ZEAMMB73_Zm00001d003257	24	8.8	3	3	1	1.71	0.00012	0.80	0.00512	0.47	0.0000153
B6TBM1	Alpha-soluble NSF attachment protein OS = Zea mays GN = ZEAMMB73_Zm00001d049982	119	39.1	9	10	9	1.41	0.00308	0.78	0.000221	0.55	0.000385
B6TDB5	17.4 kDa class I heat shock protein 3 OS = Zea mays GN = ZEAMMB73_Zm00001d028555	33	18.4	3	5	3	0.56	4.2E-06	0.64	0.00132	1.14	0.0887
B6TRV8	ARM repeat superfamily protein OS = Zea mays GN = ZEAMMB73_Zm00001d004676	13	28.5	11	15	1	1.15	0.0159	0.70	0.000737	0.61	0.000198
B6TT66	Ribosome-like protein OS = Zea mays GN = ZEAMMB73_Zm00001d015628	41	28.1	4	6	3	1.39	0.0119	0.88	0.203	0.64	0.00216
B6U284	14–3-3-like protein OS = Zea mays GN = ZEAMMB73_Zm00001d050375	48	43	10	13	3	1.84	0.00022	1.03	0.611	0.56	0.00082
B6U581	Ribosome-like protein OS = Zea mays GN = ZEAMMB73_Zm00001d053713	57	42.4	6	10	5	1.35	0.00298	0.78	0.00218	0.58	0.00046
B6UG10	40S ribosomal protein S3a OS = Zea mays GN = ZEAMMB73_Zm00001d048157	85	35.8	10	18	10	1.34	0.0001	0.76	0.000324	0.57	0.0000355
B6UIC1	50S ribosomal protein L12–1 OS = Zea mays GN = ZEAMMB73_Zm00001d043972	181	56.3	10	43	3	1.33	0.00082	0.82	0.00558	0.62	0.0000351
C0HF19	Nucleosome assembly protein 1 OS = Zea mays GN = ZEAMMB73_Zm00001d045352	7	10	2	2	1	1.72	4.2E-05	0.84	0.0165	0.49	0.000622
C0P531	26S proteasome non-ATPase regulatory subunit 3 homolog A OS = Zea mays GN = ZEAMMB73_Zm00001d053232	96	21.8	9	10	9	1.21	0.0251	0.72	0.00000138	0.60	0.0000803
C0PDG3	Heat shock protein 90–6 mitochondrial OS = Zea mays GN = ZEAMMB73_Zm00001d041719	162	20.8	13	16	8	1.45	0.00086	0.73	0.0189	0.51	0.0016
C4JBB8	Heat shock 70 kDa protein 9 mitochondrial OS = Zea mays GN = ZEAMMB73_Zm00001d006036	64	22.3	14	19	9	1.22	0.00146	0.62	0.00000132	0.51	0.0000168
K7TTX0	Plant UBX domain-containing protein 4 OS = Zea mays GN = ZEAMMB73_Zm00001d025628	31	11.8	3	4	1	1.11	0.666	0.63	0.00688	0.57	0.0355
K7U5A5	14–3-3-like protein OS = Zea mays GN = ZEAMMB73_Zm00001d053090	193	59.7	16	22	11	1.42	0.00012	0.87	0.0101	0.61	0.0000389
K7VEB9	Importin subunit alpha OS = Zea mays GN = ZEAMMB73_Zm00001d009850	29	37.8	14	22	2	1.41	0.0209	0.93	0.452	0.66	0.0037
O24415	60S acidic ribosomal protein P2B OS = Zea mays GN = RPP2B	7	10.6	1	2	1	1.37	0.00026	0.90	0.00862	0.65	0.0000431
O64960	23.6 kDa heat shock protein mitochondrial OS = Zea mays GN = hsp22	18	9.2	2	2	2	0.65	0.00094	0.63	0.00222	0.98	0.719
P24631	17.5 kDa class II heat shock protein OS = Zea mays	43	30.4	3	4	3	0.52	4E-05	0.58	0.0000602	1.11	0.00508

## Discussion

Plants are continuously exposed to environmental fluctuations, thus, they are facing great challenges to adapt themselves to variable environmental conditions, especially drought, waterlogging, cold, heat and other unfavorable conditions. To a certain extent, plant oxidative state could reflect the plant capability to resist biotic or abiotic stresses [33–35]. The generation and scavenging of ROS is one of plant defense mechanism to abiotic stresses. When plant is facing environmental stresses, ROS with high chemical activities are produced which could activate plant defense mechanism. However, excessive ROS would cause great damage to biological macromolecules such as proteins, nucleic acids and lipids, thus affecting their normal physiological and biochemical functions [33, 36]. In addition, fatty acid metabolism plays an important role in plant defense by maintaining the integrity of the cell membranes. And, proteins metabolism plays an important role in plant defense by governing a series of plant defense activities [37, 38]. Application of 6-BA and proteomic analysis enables us to obtain deeper insight into plant defense mechanism of summer maize to waterlogging. In this study, we found 6-BA improved waterlogged defense system by regulating a number of the proteins involved in ROS scavenging system, fatty acid and protein metabolism processes.

### ROS scavenging system activity

Accumulation of excessive ROS is an anticipated common damage under stress conditions, which could also cause serious damages to organelles such as chloroplasts, mitochondria and plasma membranes [39]. Initially, ROS accumulation stimulated protective mechanisms by initiating signal cascades in vivo. However, when ROS are accumulated to a certain high level, they will attack proteins in various manners, leading to modification of proteins such as peroxidation of amino acid residues and oxidation of sulfur groups. As a result, the structure and function of proteins are changed, and ultimately resulting in distortion of cell structures, and acceleration of plant senescence. Previous studies have shown that ROS could cause membrane peroxidation injury along with a series of damages on plant growth, such as chlorophyll degradation, aging acceleration, and impaired photosynthesis [40–42]. Protective enzymes including SOD, POD and CAT as well as ascorbic acid, glutathione such antioxidants which play functions in scavenging ROS are employed to mitigate the oxidative damage and protect cells. Peroxidases participate in the reduction of ROS by catalyzing the redox reaction of H_2_O_2_ with various hydrogen donors [43]. Because there are many genes encoding peroxidases in plant cells, numerous types of peroxidase have important roles in plant stress resistance. For example, peroxidase 21 and peroxidase 42 played a significant role in cucumber plants response to waterlogging stress [44]. And, ascorbic acid peroxidase in the cytoplasm or combined with the cell wall played key roles in ascorbic acid–glutathione cycle, acting as a terminal oxidase. Ascorbic acid peroxidase, which was regulated by various signal molecules, could efficiently scavenge excess H_2_O_2_ [45, 46].

This study showed that peroxidase (A0A1D6E530, B4FBH0, B4FKV6, B4FVT1, and C0HHA6) were up-regulated in the V3–6 and down-regulated in V3–6-B compared with CK. Although, down-regulation of L-ascorbate peroxidase (A0A1D6QMU5), Glutathione peroxidase (C0P3R8) and APx3-Peroxisomal Ascorbate Peroxidase (B6TM55) were observed in V3–6 compared with CK, no significant up-regulation of these proteins were observed in V3–6-B compared with V3–6. However, the activity of SOD, POD and CAT were significantly decreased and the contents of MDA and H_2_O_2_ were increased greatly in V3–6, compared with CK. And, these enzymes activities were increased and the MDA, H_2_O_2_ contents were effectively reduced by application of 6-BA, compared to those of waterlogging treatment. These results suggested that there may exist some additional complex mechanisms to regulate the ROS scavenging system activity besides regulating the protein expression level.

### Fatty acid metabolism

**Fig. 7:**
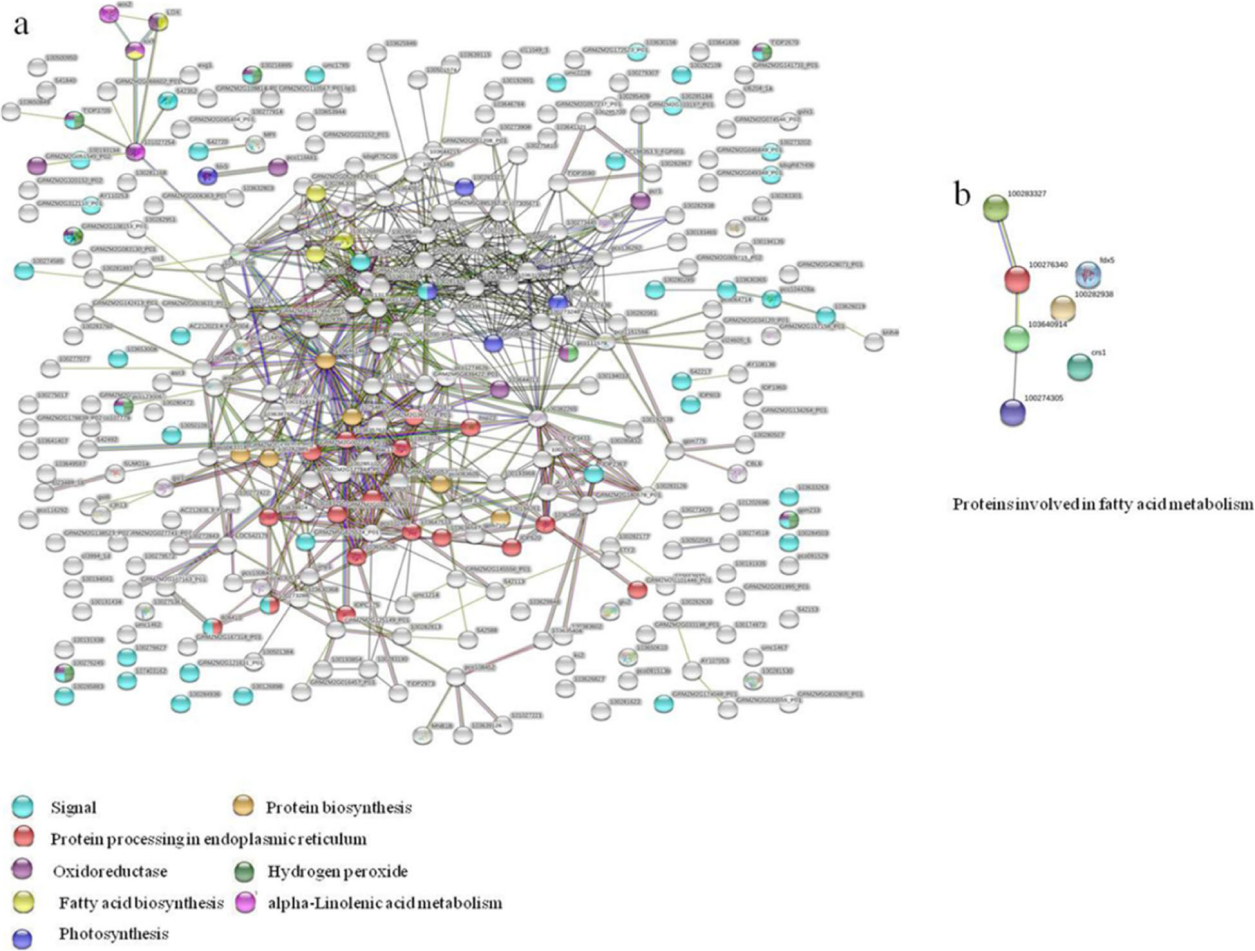
The protein-protein interaction network of the differentially abundant proteins, **a** The protein-protein interaction network of the overall differentially abundant proteins, **b** Proteins involved in fatty acid metabolism

Maintenance of membrane integrity under stress broadly reflected intrinsic tolerance [47]. Regarding fatty acid metabolism, alpha-dioxygenase 1 (A0A1D6P493 and H9BG22) and cytochrome P450 CYP74A19 (A0A096PQR7) were upregulated in both the V3–6 and V3–6-B treatments, and increased more in V3–6-B than in V3–6. In addition, lipoxygenase (Q9LKL4) also showed an increase in both V3–6 and V3–6-B. However, the magnitude of the increase was significantly higher in V3–6 than in V3–6-B, while, lipoxygenase (A0A1D6JQF2) and acyl carrier protein (B4FDG2 and B6SJF5) were up-regulated in V3–6 and down-regulated in V3–6-B. Plant alpha-dioxygenases convert fatty acids to 2-hydroperoxy products which plays an important role in plant signaling pathways [48]. Cytochrome P450s exist ubiquitously in all organisms playing important roles in many biological processes. For example, Cytochrome P450 A2 plays a key role in signal and defense reactions in higher plants [49]. The up-regulation of these two enzymes in V3–6 and V3–6-B treatments revealed that plant employed a positive response mechanism to address waterlogging stress. Acyl carrier protein (B4FDG2 and B6SJF5), lipoxygenase (A0A1D6JQF2 and Q9LKL4) and cytochrome P450 CYP74A19 (A0A096PQR7) were enriched in fatty acid biosynthesis pathway. The protein-protein network of interactions showed that lipoxygenase (A0A1D6JQF2) might bind with cytochrome P450 CYP74A19 (A0A096PQR7) to perform their functions. In addition, lipoxygenase (A0A1D6JQF2 and Q9LKL4) and cytochrome P450 CYP74A19 (A0A096PQR7) were also enriched in alpha-Linolenic acid metabolism. Furthermore, lipoxygenase (A0A1D6JQF2 and Q9LKL4) had interactions with alpha-dioxygenase 1 (A0A1D6P493 and H9BG22), which also played important roles in alpha-Linolenic acid metabolism (Fig. 7). These results showed that lipoxygenase (A0A1D6JQF2 and Q9LKL4) played a central role in both alpha-Linolenic acid metabolism and fatty acid biosynthesis pathway.

Alpha-linolenic acid, an important precursor of the cell signaling molecule jasmonic acid, could induce jasmonic acid accumulation. Also, alpha-linolenic acid appears to be necessary for biomembrane integrity and function, which plays a key role in protecting plants from environmental stress damage. Plant resistance to cold is weakened with a decrease in unsaturated acids, as shown in *Arabidopsis thaliana* by mutating the chloroplast sn − 2-palmitoyl desaturase and △12-desaturase genes [50, 51]. Increasing the expression of fatty acid-desaturating genes in plants can increase the production of alpha-linolenic acid [52, 53]. As the membrane components, particularly of phospholipids, Linoleic and alpha-Linolenic acids play key roles in maintaining membrane integrity. In addition, they are also substrates for lipoxygenase (LOX). Therefore, LOX could do destructive damages to cell membranes and other cell components [54]. An increase in lipoxygenase always accompanied damage of unsaturated fatty acids, as lipoxygenase could oxidize unsaturated fatty acids and incur lipid peroxidation as well as a series of secondary reactions [55]. In addition, lipoxygenase reactions are also the sources of ROS [56]. Excessive LOX and ROS could induce lipid peroxidation, loss of membrane integrity which has been proved in a wide range of plants such as wheat, bean, cotton, maize and so forth. Interestingly, a close association between LOX and ROS has also been established [54].

Turning to our proteomic data, the proteins involved in alpha-Linolenic fatty acids were up-regulated in V3–6 and V3–6-B treatments. And, the magnitude of the up-regulated level was higher in V3–6-B than in V3–6. However, the lipoxygenase (A0A1D6JQF2) and other proteins (acyl carrier protein (B4FDG2 and B6SJF5)) involved in fatty acid biosynthesis pathway were up-regulated in V3–6, and down-regulated in V3–6-B. The up-regulation of proteins involved in alpha-Linolenic fatty acids might maintain the plant membrane structure to some extent. However, the increased expression of lipoxygenase (A0A1D6JQF2) might lead to damaged membrane structures and functions. In order to validate the above-mentioned results of protein abundance, the LOX activity and its byproduct (H_2_O_2_ and MDA) content were analyzed. The results indicated that LOX activity was significantly increased after waterlogging and the content of its byproducts (MDA and H_2_O_2_) were increased correspondingly which hinted damages on membrane structure incurred by waterlogging. However, the activity of LOX and the content of H_2_O_2_ and MDA were effectively reduced after application of 6-BA which supported the results attained by labeling liquid chromatography-based quantitative proteomic analysis and LC-MS/MS. Overall, the present study proved that the waterlogged summer maize plant stimulated some positive response strategies to address waterlogging stress. However, the waterlogging damages were also unequivocally confirmed. Application of 6-BA was conducive to upregulate the proteins (alpha-dioxygenase 1 (A0A1D6P493 and H9BG22) and cytochrome P450 CYP74A19 (A0A096PQR7)) involved in positive response mechanisms of waterlogged plant and downregulate the proteins (LOXs) which do damages on plants. The physiology results (decreased activity of LOX and content of H_2_O_2_ and MDA in V3–6-B) further proved that 6-BA played a pivotal role in reducing the production of ROS by regulating the proteins expression involved in fatty acid metabolism. Spraying 6-BA was conducive to protect the plant membrane from peroxidation damage to some extent.

### Proteins involved in protein metabolism

Protein metabolism processes are closely associated with plant growth and development by regulating a series of biological activities [57]. Zinc finger protein is a transcriptional regulatory factor that regulates gene expression by binding zinc and iron centers to DNA [58, 59], which modulates plant growth and development by regulating gene expression [60, 61]. It also plays a very important role in plant response to biotic and abiotic stresses [59, 62–64]. In rice, up-regulation of two zinc finger proteins (Q67TK9, Q10N88) and down-regulation of another zinc finger protein (Q5YLY5) contributes to increases of heat tolerance duration at night during grain filling. Our results showed that the RING/FYVE/PHD zinc finger superfamily protein (A0A1D6GZM9) was increased in V3–6 treatment and decreased in V3–6-B treatment which might relate with waterlogging tolerance of summer maize. In addition, MBF1 transcription factor (C0P5I3) and NmrA-like negative transcriptional regulator family protein (A0A1D6L6U7) were decreased in both V3–6 and V3–6-B. However, the magnitude of decrease was higher in V3–6 than in V3–6-B. MBF1 transcription factor was a transcriptional Co-activator MBF1c that has a function in thermo-tolerance of *Arabidopsis thaliana* [65].

NmrA, a negative transcriptional regulator, has function in controlling nitrogen metabolite repression in various fungi by modulating AreA, a kind of GATA-type transcription factor. And, Reiner et al. (2016) proved that a new protein with an NmrA-like domain is involved in cell differentiation and development of Dictyostelium discoideum [66, 67]. These results suggested that gene involved in stress response or cell development processes were significantly down regulated in V3–6 which might be responsible for the intolerance to waterlogging of summer maize. However, Compared with CK, proteins involved in translation process, such as nucleosome assembly protein 1 (C0HF19 and A0A1D6MBB0), elongation factor 1-beta (B6SLK4 and B4FNT1), 30S ribosomal protein S1 (B4FUZ5), 60S ribosomal protein L22–2 (B4FCK9), and nucleic acid-binding OB-fold-like proteins (A0A1D6H4K9 and B4FT80) were significantly increased in V3–6, while spraying 6-BA was effectively conducive to mitigate these increases.

Most proteins are required to fold into fine three-dimensional structures to perform functional activity. However, many proteins fold aberrantly and even aggregated under stress conditions [68, 69]. Protein aberrant folding and aggregation occurred with an increasing frequency under environmental stresses such as high temperature and drought [70]. Therefore, plant needs to evolve numerous mechanisms to address aberrant folding and aggregation. Hsp90 and small Hsps have been demonstrated to have functions in preventing aggregation and promoting efficient folding under adverse conditions [71]. Recent research also demonstrated that drought aroused a remarkable increase of some sHSPs in maize leaves [72]. In our study, Hsps (O64960, B6TDB5, and P24631) were significantly down-regulated in waterlogged plant leaves, while 6-BA mitigated these decreases. However, the key constituents of the degradation system, 26S proteasome subunits (A0A1D6HCE3, B6T9G1, A0A1D6KA58, and A0A1D6GTD7), were up-regulated in waterlogged plants whereas no significant changes were observed in the V3–6-B treatment. In addition, proteins associated with unfolded protein binding, phosphatase, proteolysis, and ubiquitination (A0A1D6E942 and B4FUZ5; B6UG10; A0A1D6H5B6, B4FKB3) were decreased greatly in V3–6. These results suggested waterlogging affected the protein folding and other processing processes which impeded the proteins to perform functions. However, application of 6-BA was conducive to mitigate waterlogging impacts on these proteins.

These results indicated that waterlogging triggered notable changes in protein metabolism process including protein synthesis, protein processing, protein homeostasis and degradation processes. The extensive changes in protein metabolism process might result in disorders of cell metabolism activities such as substance synthesis, transport, and secondary metabolism processes. In our previous study, carbon and nitrogen metabolism processes were significantly affected by waterlogging, which disturbed the balance of cell activities, affecting substance synthesis and transport, and thus limited plant growth. Impediment of plant growth induced by waterlogging was also observed in this study, while spraying 6-BA mitigated this impediment [26, 73].

Overall, the present study proved that 6-BA increased the defense system activity by modulating the expression of proteins related with ROS and fatty acid metabolism which worked together to maintain the integrity of cell membranes. Conversely, the increased integrality of cell membranes improved the protein metabolism processes which might be responsible for the improvement of plant growth rate and yield of waterlogged summer maize.

## Conclusions

**Fig. 8:**
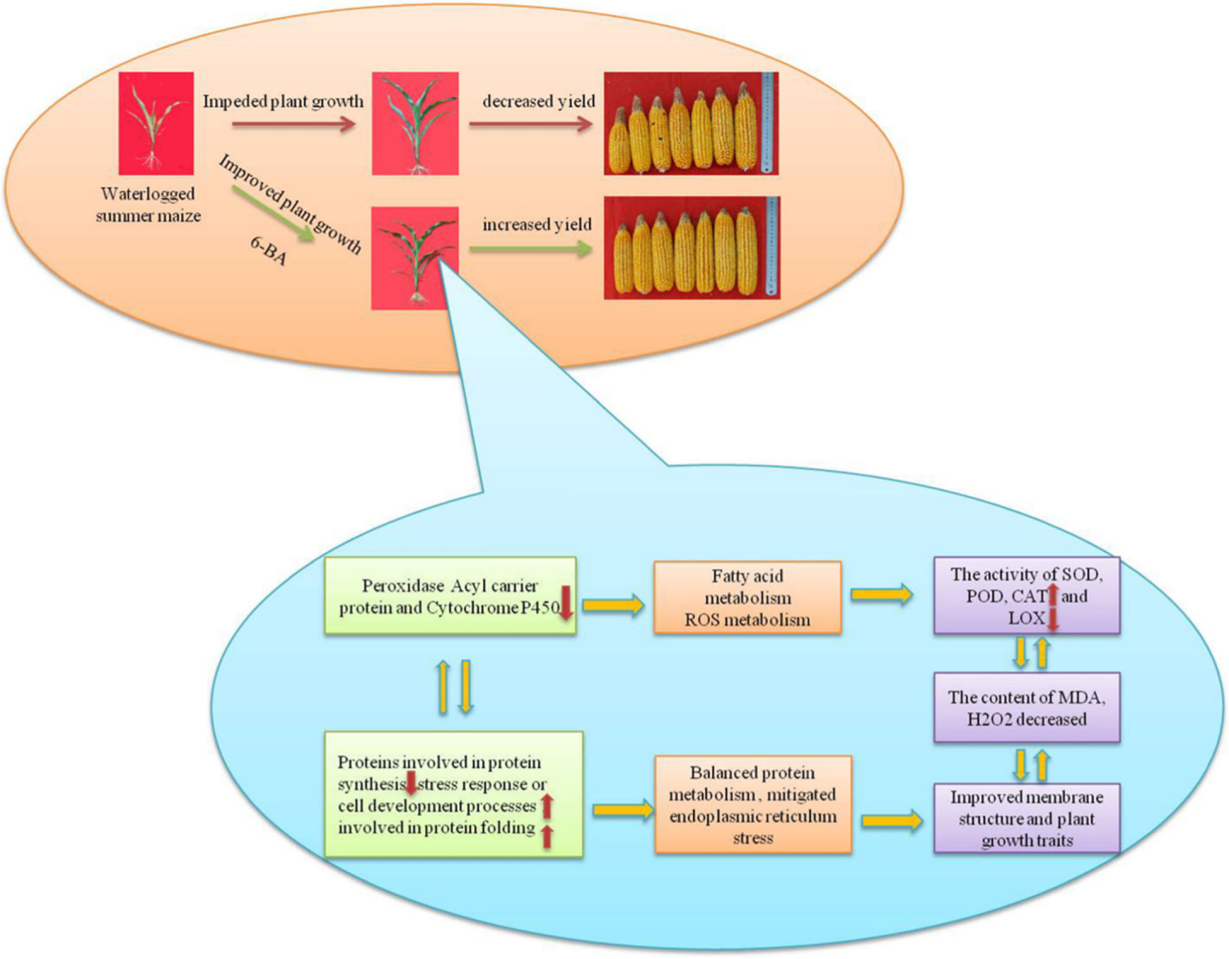
Exogenous 6-benzyladenine (6-BA) improve the defense system activity of waterlogged summer maize

Based on our study, we demonstrated that 6-BA had contrastive effects on waterlogged summer maize (Fig. 8). Waterlogging caused significant impediment of plant growth and decreased the activities of SOD, POD and CAT of summer maize. In addition, the activity of LOX was significantly increased. As a result, the content of MDA and H_2_O_2_ was significantly increased which incurred serious damages on cell membrane and cellular metabolism. In addition, the plant growth rate and grain yield were significantly decreased by waterlogging. However, application of 6-BA effectively mitigated these adverse effects induced by waterlogging. Based on proteomics profiling, the proteins involved in protein metabolism, ROS scavenging and fatty acid metabolism were significantly regulated by 6-BA. The results suggested that application of 6-BA exaggerated the defensive response of summer maize at proteomic level leading to improved plant growth traits and a higher grain yield. Although further studies are required to investigate the regulatory mechanism of 6-BA on waterlogged summer maize, the results in our study provide a foundation for further researches.

## Methods

### Experimental design

The field experiment was performed at the experimental farm of Shandong Agricultural University, China (36° 10′ N, 117° 09′ E). This study was conducted in accordance with local legislations and obtained the permission from Shandong Agricultural University complying with the Convention on the Trade in Endangered Species of Wild Fauna and Flora. The summer maize hybrid DengHai605 (DH605) was used in this study. DH605 is the hybrid of DH351 (selected from a self-breeding multiple generation of DH158/107) and DH382 (selected from a self-breeding multiple generation of American germplasm X1132) bred by Shandong Denghai Seeds Co., Ltd. It was approved by the National crop variety examination and approval committee of china on 09^th^ 2010 which suitable for planting in Shandong province and most part of china. Maize was sown on June with a plant density of 67,500 plants ha^− 1^ and well managed. Three experimental treatments were set: control (CK), waterlogging at the third leaf stage for 6 days (V3–6) and 100 mg dm^− 3^ 6-BA was applied after waterlogging (V3–6-B). Three replicated fresh ear leaf tissue were sampled at tasseling stage from each treatment and frozen in liquid nitrogen and kept at − 80 °C until further use. No permissions or licenses were needed to obtain our plant sample.

### Physiological traits determination

#### Antioxidant enzyme activity and MDA content

The middle of functional leaf from three plant samples was obtained to determine the protective enzymes activity and MDA content. The activity of SOD, POD, and CAT was assayed according to the method described by Giannopolitis and Ries (1977), Hammerschmidt et al. (1982) and Durner and Klessing (1996), respectively [74–76]. In addition, the MDA content was determined according to Du et al. (1992) [77]. Three biological replicates of the individual samples were used for analysis. Oneway ANOVA and comparisons between means were made following the LSD test at *P* < 0.05 and performed with SPSS V.21.0.

#### Estimation of LOX activity and H_2_O_2_ content

The LOX activity and H_2_O_2_ content were determined according to the method described by Fu et al. (1996) [78] and Bizzi et al. [79], respectively. One-way ANOVA and comparisons between means were made following the LSD test at *P* < 0.05 and performed with SPSS V. 21.0.

#### Plant growth rate and yield

The number of the fully extended leaves was observed at V6, V9, V12 growth stages (based on 50% of all plants in the plot attaining the growth stage) of CK. Fifteen representative plant height was measured at VT. Thirty ears from each plot were harvested to calculate yield at R6. One-way ANOVA and comparisons between means were made following the LSD test at *P* < 0.05 and performed with SPSS V. 21.0.

### Proteomic analysis

The leaf samples of three plants from each treatment were mixed and exposed to liquid nitrogen, ground into cell powder and then four volumes of lysis buffer (8 M urea, 1% Protease Inhibitor Cocktail) were added to the cell powder for extracting proteins. Then, the extracted protein solutions were digested by trypsin. After digestion, the peptides were labeled by TMT then for HPLC fractionation using Thermo Betasil C18 column (5 μm particles, 10 mm ID, 250 mm length; Bellefonte, PA, USA) and LC-MS/MS analysis using Orbitrap FusionTM (ThermoFisher Scientific, Waltham, USA). The MS/MS data were searched against UniProt *Zea mays* L database (99,450 sequences, 201,706) concatenated with reverse decoy database using the Maxquant search engine (v.1.5.2.8). Trypsin/P was specified as a cleavage enzyme allowing up to 2 missing cleavages. The mass tolerances for precursor ions in First search and Main search were set as 20 ppm and 5 ppm, respectively. 0.02 Da was set as the mass tolerance for fragments. Carbamidomethyl on Cys and oxidation on Met (or Acetylation on the N-terminal) were specified as fixed modification and variable modifications, respectively. False discovery rate (FDR) was set < 1% and minimum score for peptides was set > 40 to assess the confidence of the peptides [80]. TMT 10-plex was used in Mascot to quantify identified proteins. And, proteins with *P*-value < 0.05 and fold changes > 1.5 or < 0.667 were considered as significant differentially abundant. After screening for differentially abundant multiples, bioinformatics analysis including protein annotation, functional classification, functional enrichment, and cluster analysis of differentially abundant proteins were performed. The Gene Ontology functional annotations (GO) and Kyoto Encyclopedia of Genes and Genomes analysis (KEGG) of identified proteins were searched against GO database (www.http://www.ebi.ac.uk/GOA/) and KEGG database (http://www.genome.jp/kegg/pathway.html) using Blast2GO program (https://www.blast2go.com/) and Blastx/Blastp 2.2.24 software, respectively. Wolfpsort (an updated version of PSORT/PSORT II) was used to predict subcellular localization. Then, GO and KEGG pathway enrichment analysis of the DAPs were implemented with a *P*-value < 0.05 [81]. Three technical replicates of each sample were performed. This proteomic analyses was provided by Jingjie PTM BioLabs, Inc. The more detailed method was described in Additional file 4.

## Supplementary information

**Additional file 1: **Quality detection of mass spectrometry and part of results, **Figure S1.** The distribution of peptide mass error and peptide length. **Figure S2.** The Pearson’s correlation of quantitation among treatments. **Figure S3.** The differentially abundant proteins among treatments.

**Additional file 2.** GO term level2 classification of differentially abundant proteins.

**Additional file 3.** Details of differentially abundant proteins.

**Additional file 4.** Details of MS Methods.

## Data Availability

The data supporting the conclusions of this article are included within the article and its additional files. And also, the mass spectrometry protemics datasets have been deposited to the ProteomeXchange Consortium via the PRIDE [82] partner repository with the dataset identifier PXD016743”, (https://www.ebi.ac.uk/pride/profile/reviewer85471).

## Abbreviations

6-BA: 6-benzyladenine
ANOVA: Analysis of variance
CAT: Catalase
CK: Control
Cys: Cysteine
DAPs: Differentially abundant proteins
DH605: Summer maize hybrid DengHai 605
FDR: False discovery rate
GO: Gene Ontology
H_2_O_2_: Hydrogen peroxide
HPLC: High-pressure liquid chromatography
KEGG: Kyoto Encyclopedia of Genes and Genomes
LC: Liquid chromatography
LOX: Lipoxygenase
LSD: Least significant difference
MDA: Malondialdehyde
MS: Mass spectrometry
POD: Peroxidase
ROS: Reactive oxygen species
SOD: Superoxide dismutase
TEAB: Tetraethylammonium bromide
TMT: Tandem Mass Tags
V12: Twelfth leaf stage
V3–6: Waterlogging at the third leaf stage for 6 days
V3–6-B: Application of 100 mg dm^− 3^ 6-BA after waterlogging for 6 days
V6: Sixth leaf stage
V9: Ninth leaf stage
VT: Tasseling stage
