# Supplementary material for: Comparative proteomic analysis reveals that exogenous 6-benzyladenine (6-BA) improves the defense system activity of waterlogged summer maize
Source: BMC Plant Biol. 2020 Jan 29;20:44. doi: 10.1186/s12870-020-2261-5 (PMC6988316; doi:10.1186/s12870-020-2261-5)
Supplement: Supplementary file 1 — Additional file 1: Quality detection of mass spectrometry and part of results, Figure S1. The distribution of peptide mass error and peptide length. Figure S2. The Pearson’s correlation of quantitation among treatments. Figure S3. The differentially abundant proteins among treatments. [file 12870_2020_2261_MOESM1_ESM.docx]

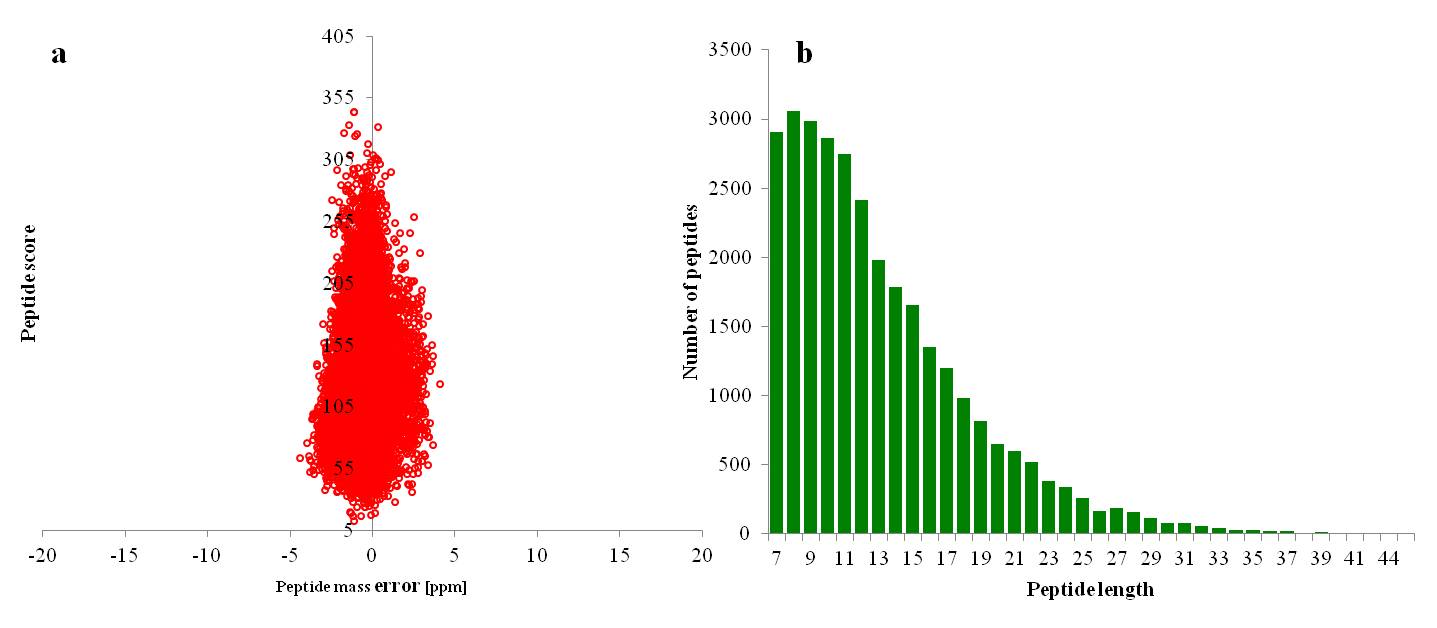


Figure S1 The distribution of peptide mass error and peptide length.


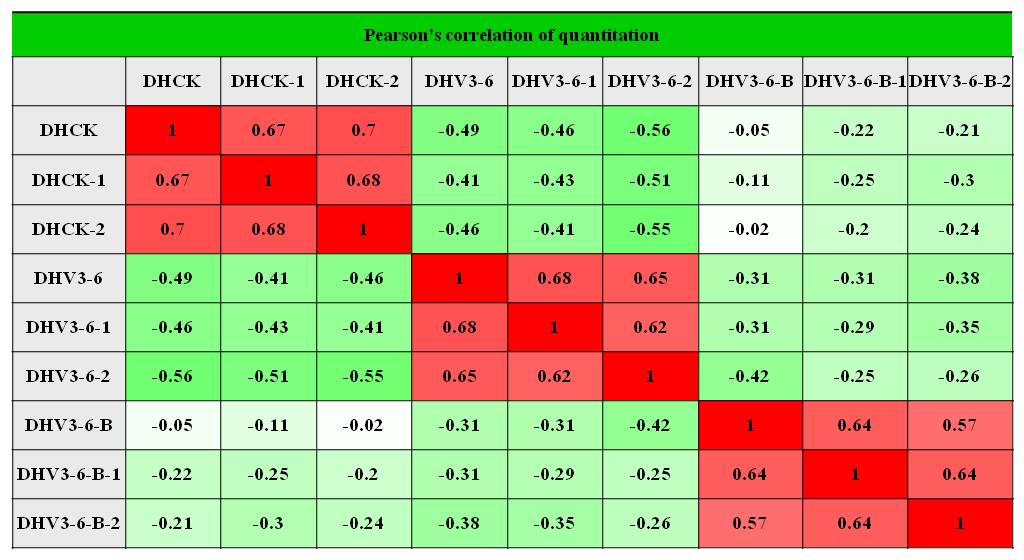


Figure S2 The Pearson’s correlation of quantitation among treatments.


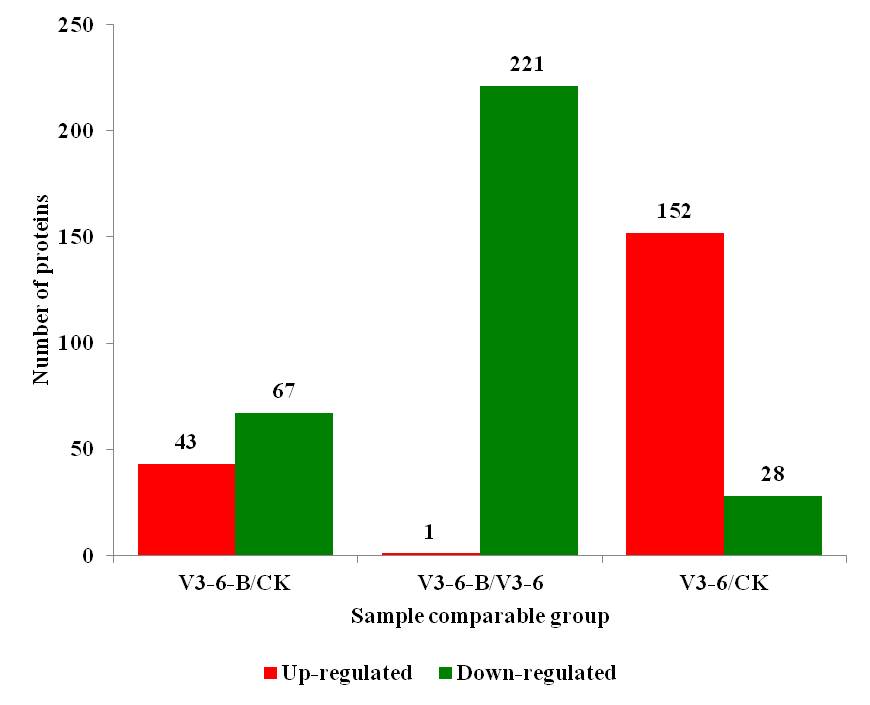


Figure S3 The differential abundant proteins among treatments.
