# Supplementary material for: Comparative proteomic analysis reveals that exogenous 6-benzyladenine (6-BA) improves the defense system activity of waterlogged summer maize
Source: BMC Plant Biol. 2020 Jan 29;20:44. doi: 10.1186/s12870-020-2261-5 (PMC6988316; doi:10.1186/s12870-020-2261-5)
Supplement: Supplementary file 4 — Additional file 4. Details of MS Methods. [file 12870_2020_2261_MOESM4_ESM.docx]

**Proteomic analysis**

**Protein Extraction**

The sample was exposed to liquid nitrogen, ground into cell powder and then transferred to a 5-mL centrifuge tube. After this, four volumes of lysis buffer (8 M urea, 1% Protease Inhibitor Cocktail) were added to the cell powder, followed by sonication three times on ice using a high intensity ultrasonic processor (Scientz). The remaining debris was removed by centrifugation at 12,000 × g at 4 °C for 10 min. Finally, the supernatant was collected and the protein concentration was determined with (BCA) kit according to the manufacturer’s instructions.

**Trypsin Digestion**

For digestion, the protein solution was reduced with 5 mM dithiothreitol for 30 min at 56 °C and alkylated with 11 mM iodoacetamide for 15 min at 25 °C in darkness. The protein sample was then diluted by adding 100 mM Tetraethylammonium bromide (TEAB) to urea concentration less than 2 M. Finally, trypsin was added at 1:50 trypsin-to-protein mass ratio for the first digestion overnight and 1:100 trypsin-to-protein mass ratio for a second 4 h-digestion.

**TMT Labeling**

After trypsin digestion, peptide was desalted by a Strata X C18 SPE column (Phenomenex, Torrance, CA, USA) and vacuum-dried. Peptides were reconstituted in 0.5 M TEAB and processed according to protocols from the manufacturer for the TMT kit (AB Sciex, Foster City, CA). Briefly, one unit of TMT reagent was thawed and reconstituted in acetonitrile. The peptide mixtures were then incubated for 2 h at 25 °C, pooled, desalted, and dried by vacuum centrifugation.

**HPLC Fractionation**

The tryptic peptides were fractionated by high pH reverse-phase HPLC using Agilent 300Extend C18 column (5 μm particles, 4.6 mm ID, 250 mm length). Briefly, peptides were first separated with a gradient of 8% to 32% acetonitrile (pH 9.0) over 60 min into 60 fractions. Then, the peptides were combined into 18 fractions and dried by vacuum centrifuging.

**LC-MS/MS Analysis**

The tryptic peptides were dissolved in 0.1% formic acid (solvent A), directly loaded onto a home-made reversed-phase analytical column (15 cm length, 75 μm i.d.). The gradient was comprised of an increase from 6% to 23% solvent B (0.1% formic acid in 98% acetonitrile) over 26 min, 23% to 35% in 8 min and climbing to 80% in 3 min then holding at 80% for the last 3 min; all at a constant flow rate of 400 nL/min on an EASY-nLC 1000 UPLC system.

The peptides were subjected to NSI source followed by tandem mass spectrometry (MS/MS) in Q Exactive^TM^ Plus (Thermo) coupled online to the UPLC. The electrospray voltage applied was 2.0 kV. The m/z scan range was 350 to 1800 for full scan, and intact peptides were detected in the Orbitrap at a resolution of 70,000. Peptides were then selected for MS/MS using NCE setting as 28 and the fragments were detected in the Orbitrap at a resolution of 17,500. A data-dependent procedure that alternated between one MS scan followed by 20 MS/MS scans with 15.0 s dynamic exclusion. Automatic gain control (AGC) was set at 5E4. The fixed first mass was set as 100 m/z.

**Database Search**

The resulting MS/MS data were processed using the Maxquant search engine (v.1.5.2.8). Tandem mass spectra were searched against UniProt Zea mays L database concatenated with reverse decoy database. Trypsin/P was specified as a cleavage enzyme allowing up to 2 missing cleavages. The mass tolerance for precursor ions was set as 20 ppm in First search and 5 ppm in Main search, and the mass tolerance for fragment ions was set as 0.02 Da. Carbamidomethyl on Cys was specified as fixed modification and oxidation on Met was specified as variable modifications. FDR was adjusted to < 1% and minimum score for peptides was set > 40. Quantitative protein ratios were weighted and normalized by the median ratio in Mascot. A protein with Bonferroni-corrected P-value < 0.05 and fold changes > 1.5 or < 0.667 was considered as being significant differentially expressed in the pairwise comparison.

Gene Ontology (GO) (www. <http://www.ebi.ac.uk/GOA/>) functional annotation of identified proteins were searched against the non-redundant protein database using Blast2GO program (https://www.blast2go.com/). Kyoto Encyclopedia of Genes and Genomes (KEGG) (http://www.genome.jp/kegg/pathway.html) database was adopted to categorize these identified protein species using Blastx/Blastp 2.2.24 software. Wolfpsort an updated version of PSORT/PSORT II was used to predict subcellular localization. Then, GO and KEGG pathway enrichment analysis of the DEPs were implemented with a P-value < 0.05. Statistical analyses were conducted using analysis of variance (ANOVA) in SPSS 18.0. We assessed differences among treatments using a least significant difference (LSD) test at a probability level of 0.05.
